# Spatial Genetic Analyses Reveal Cryptic Population Structure and Migration Patterns in a Continuously Harvested Grey Wolf (*Canis lupus*) Population in North-Eastern Europe

**DOI:** 10.1371/journal.pone.0075765

**Published:** 2013-09-19

**Authors:** Maris Hindrikson, Jaanus Remm, Peep Männil, Janis Ozolins, Egle Tammeleht, Urmas Saarma

**Affiliations:** 1 Department of Zoology, Institute of Ecology and Earth Sciences, University of Tartu, Tartu, Estonia; 2 Estonian Environment Information Centre, Tartu, Estonia; 3 State Forest Research Institute “Silava,” Salaspils, Latvia; Université de Sherbrooke, Canada

## Abstract

Spatial genetics is a relatively new field in wildlife and conservation biology that is becoming an essential tool for unravelling the complexities of animal population processes, and for designing effective strategies for conservation and management. Conceptual and methodological developments in this field are therefore critical. Here we present two novel methodological approaches that further the analytical possibilities of STRUCTURE and DResD. Using these approaches we analyse structure and migrations in a grey wolf ( *Canis* *lupus* ) population in north-eastern Europe. We genotyped 16 microsatellite loci in 166 individuals sampled from the wolf population in Estonia and Latvia that has been under strong and continuous hunting pressure for decades. Our analysis demonstrated that this relatively small wolf population is represented by four genetic groups. We also used a novel methodological approach that uses linear interpolation to statistically test the spatial separation of genetic groups. The new method, which is capable of using program STRUCTURE output, can be applied widely in population genetics to reveal both core areas and areas of low significance for genetic groups. We also used a recently developed spatially explicit individual-based method DResD, and applied it for the first time to microsatellite data, revealing a migration corridor and barriers, and several contact zones.

## Introduction

Anthropogenic activities are among the key factors affecting wildlife populations, and perhaps most important among them are overexploitation and habitat destruction/fragmentation, which cause a considerable range of problems not only for wildlife, but for sustainable development in general (e.g. [1-3]). These factors are also important in shaping the spatial population processes of mammals and in altering their population structure and distribution patterns. Therefore, understanding the effects of anthropogenic activities is becoming increasingly important for the development of effective conservation and management strategies. The relatively new field of spatial genetics, or ’landscape genetics’ [4], uses population genetic and spatial data to study interactions between the spatial patterns of populations and ecological factors, the latter inevitably including anthropogenic factors.

Highly mobile species such as wolf, brown ( *Ursus* *arctos* ) and black bear (*U. americanus*) make suitable study species for investigating large-scale spatial and temporal population processes in large carnivores. Spatial genetic analyses have demonstrated, for example, how results from population viability analyses of Mexican wolf ( *Canis* *lupus* *baileyi*) can be combined with habitat data to develop quantitative recovery criteria for population connectivity [5]; they have also revealed important geographic mixing areas for different brown bear subpopulations [6], cryptic brown bear phylogeographical patterns [7], and have demonstrated the impacts of anthropogenic forces on the spatial genetic structure of black bear populations [8]. Although a set of methodological approaches have been developed in spatial genetics over the last decade (reviewed in 9), the field would benefit from further conceptual and methodological advancement.

A growing body of evidence suggests that overexploitation has had severe consequences for many wildlife populations and has driven a number of species to extinction [10]. Wildlife conservation and management decisions have traditionally been executed on the basis of data that relate to demographic factors affecting the abundance and growth rates of protected or intensively managed populations. Although the effects of hunting on wild animal populations are quite well known [11,12], most of them are often ignored by managers. In addition to reduction in population size and density, which are usually considered, severe hunting pressure can lead also to population fragmentation, increased immigration from other populations, disruption of social systems (e.g. [12]), and can even increase the rate of hybridization with closely related species (e.g. [13]). It can also lead to higher juvenile mortality and increased immigration, as shown for grey wolf ( *Canis* *lupus* ) [14] and for cougar ( *Puma* *concolor* ) [15]. The life-history changes experienced by species subject to hunting strongly suggest that intensive harvest can induce evolutionary responses in wild populations [16,17]. For species such as wolves that exhibit a kin-based social structure, the preservation of family groups is of evolutionary significance, as fitness is positively associated with the maintenance of sociality [18-23]. Moreover, mating system and sociality influence fine-scale genetic structure via patterns of breeding and pack formation, and influence overall population structure by shaping dispersal and gene flow [24-26].

Grey wolves are capable of adapting to a wide range of ecological conditions. Recent evidence suggests that the social organisation of wolves into packs might be one reason explaining the evolutionary success of the species; packs enable wolves to effectively use a wide range of resources to feed and guarantee better survival of their young [27-29]. Under natural conditions, i.e. in the absence of strong hunting pressure, wolves generally live in kin-based packs containing a dominant pair of adults, their offspring and close relatives. When the offspring mature, they often disperse and live solitarily for a period before finding a mate and territory and producing offspring. Packs are usually nomadic within territories [30]. Severe hunting pressure can, however, break up this natural social structure into smaller entities [31] with the adoption of unrelated individuals into packs, resulting in low kinship [26,32] and sometimes territory abandonment [14] and hybridization with dogs (e.g. [13,33-35]). Although it has been proposed that wolf populations compensate for human exploitation via adjustments in dispersal, including immigration [36], a strong association has been found between human off-take and total mortality rates of wolves in North-America [37].

Following changes to public attitudes and the introduction of favourable legislation, many wolf populations in Europe have expanded in recent decades, slowly recolonizing parts of their former range (reviewed in 38-40). Throughout much of north-eastern Europe wolf populations have remained at apparently secure population levels with regulated human harvests. However, in some countries, such as Estonia and Latvia, wolf abundance and density has been considerably reduced by strong hunting activity. Wolf populations in Estonia and Latvia are believed to be part of the continuous Baltic wolf population [39] which extends through all three Baltic countries, Estonia, Latvia and Lithuania, and is connected to populations in western continental Russia, eastern Poland, northern Ukraine and Belarus. Estonian and Latvian populations went through severe demographic bottlenecks around the mid-1960s, when the estimated average population size during 1966-1970 was about 13 individuals in Latvia and nine in Estonia. Populations in both countries started to recover in the second half of 1970s and reached their maximum in the middle of 1990s, when in Estonia and Latvia the population census sizes were about 700 and 900-1000 animals, respectively. During that period, hunting pressure also escalated, with annual harvests constituting from one third to nearly half of the population census in both countries (Figure S1). Most probably as a result of the severe hunting pressure putative wolf-dog hybrids started to appear in both countries, and the hybrid status of several individuals has recently been verified with genetic analysis [34,35]. However, no studies have yet investigated the genetic composition and population structure of the wolf population in Estonia and Latvia. In addition, the wolf population in Estonia and Latvia serves as a good model for studying population structure and processes in a population that has been under high continuous hunting pressure for a considerable period of time. The current study therefore aimed to develop spatial genetic approaches to analyse population structure and patterns of gene flow in the wolf population in Estonia and Latvia, which has been under severe hunting pressure for decades.

## Materials and Methods

### Samples

**Figure 1:**
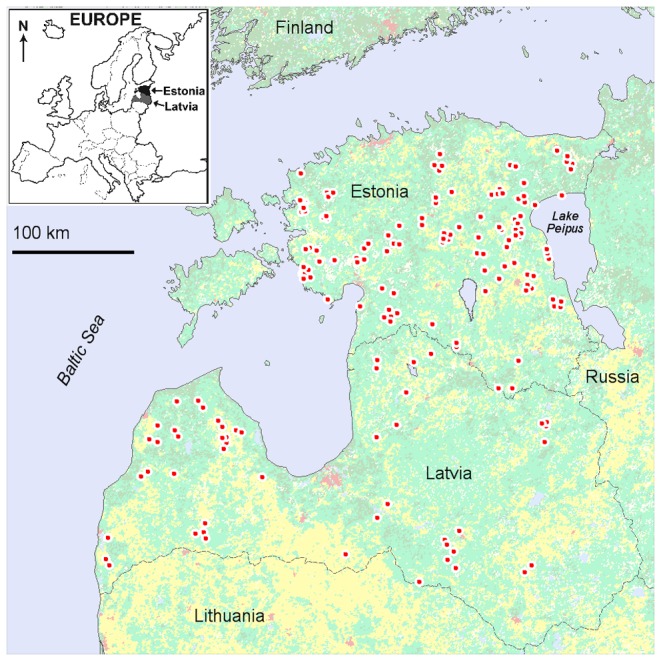
Sampling locations of wolves in Estonia and Latvia. Background colours show MODIS land cover categories: green – forests, yellow – agricultural open habitats, red – settlement, blue – waterbodies.

Wolf muscle tissue samples were collected across the species range in Estonia (n = 116) and Latvia (n = 50) between the 2004/2005 and 2008/2009 hunting seasons (Figure 1). All samples were collected from animals legally harvested by hunters for purposes other than this project. Samples were stored at -20 °C. DNA was extracted from 20-50 mg of muscle tissue using High Pure PCR Template Preparation Kit (Roche).

### Microsatellite analysis

A total of 16 autosomal microsatellite loci were analysed: FH2001, FH2010, FH2017, FH2054, FH2079, FH2088, FH2096 [41], vWF [42], AHT130 [43], M-CPH2, M-CPH4, M-CPH12 [44] and C09.173, C466, C20.253, CXX22 [45]. All loci were polymerase chain reaction (PCR) amplified in a volume of 10 microlitres containing 0.25 U Amplitaq Gold (Applied Biosystems), 1 µL of 10 × concentrated PCR buffer, 2 mM MgCl_2_, 0.2 mM dNTP, 3.3 pmol of primers and 10-50 ng of DNA. PCR reaction conditions were as follows: 10 min at 94 °C for initial denaturation, 11 cycles of 30 s at 94 °C, 30 s at 58 °C with touchdown of -0.5 °C per cycle, 1 min at 72 °C and 28 cycles of 30 s at 94 °C, 30 s at 52 °C, 1 min at 72 °C and a final elongation step for 10 min at 72 °C. After the PCR, the reaction mixture was diluted five times with water, and 0.25 µL of the molecular size standard GeneScan™ 500 LIZ (Applied Biosystems) was added to 10 µL of the dilution in order to identify the length of amplified loci. PCR products were analysed using an ABI PRISM 3100 (Applied Biosystems) automatic sequencer following the protocol provided by the manufacturer. The alleles observed for each microsatellite were sized using GENEMAPPER v4.0 (Applied Biosystems). Sample locations and microsatellite data were deposited in the Dryad Repository: http://doi.org/10.5061/dryad.2n97q.

### Estimating error rates

The presence of null alleles and stuttering were analysed with MICRO-CHECKER v2.2.3 [46]. To estimate the rate of different types of error (allelic dropout, false alleles, double and complete errors) 17 randomly chosen samples (10.2% of all samples) were blindly re-genotyped for a second time and the results were analysed using the software GIMLET v1.3.2 [47].

### Population bottlenecks

To detect the signature of a genetic bottleneck in the Estonian-Latvian wolf population, two tests in Bottleneck 1.2.02 [48,49] were performed: (1) the population was assessed for a deficiency of low frequency allele classes by examining the overall distribution of allele frequency classes (‘mode shift’ test); (2) a sign test was used to compare the number of loci that present a heterozygosity excess, to the number of such loci expected by chance only. This test is provided for three mutational models: the infinite alleles model (IAM); the stepwise mutation model (SMM); and a combination of those two extreme hypotheses, the two phase model (TPM) [50]. In the TPM, the proportion of IAM was set to 10%.

### Population structure

Bayesian assignment tests were performed with STRUCTURE v2.2 [51] to evaluate the number of genetic clusters (*K*) and to assign individuals to their likely origin. Assignment of individuals into genetic clusters was performed with STRUCTURE using five MCMC runs of 5 x 10^5^ iterations, with the first 10% of iterations discarded as burn-in. We estimated *K* using the posterior probability of the data [Ln P(D)] as suggested by Evanno et al. [52]. The initial value of alpha (Dirichlet parameter for the degree of admixture) was fixed to 1.0. We used the correlated allele frequency model implemented by Falush et al. [53], assuming that for several generations following population subdivision, the evolution of allele frequencies in each genetic group is correlated with the allele frequencies of an ancestral population and that different subpopulations have different values of *F*_*ST*_ (prior mean of *F*_*ST*_ for populations was set to 0.01). The value for λ (allele frequency parameter), which parameterizes the allele frequency prior, was kept constant and fixed to 1.0 as suggested by Pritchard et al. [51]. Factorial correspondence analysis (FCA) implemented in GENETIX v4.05.2 [54] was additionally used to investigate population sub-structuring.

The STRUCTURE documentation suggests that artificial partitions may arise due to a pattern of isolation by distance (IBD) in weakly differentiated populations. Therefore, to assess the inferred structure, we also tested the data set for IBD. We correlated the matrices of geographical distance of sample pair locations and genotype likelihood ratio distance (D_LR_), and tested for statistical significance using a Mantel test (in R package ade4 [55]). The D_LR_-index was chosen because compared with many other genetic distance indices, it performs well at fine spatial scales where individuals typically have low divergence [56].

To investigate whether genetic groups were spatially distinct, we applied an iterative linear interpolation with 1000 bootstrap permutations that we believe to be novel in the study of spatial genetics (see also 57,58). The analysis was based on the posterior probabilities (given by STRUCTURE) for individuals to belong to each of the different clusters, i.e. the expected proportions of every cluster amongst the ancestors of each sampled animal. We calculated the inverse distance weighted (w = 1/dist.) average of the probabilities from all samples for grid points spaced 5-km apart throughout the study area, as suggested by Fortin and Dale [57]. Subsequently, to estimate the ranges of the genetic groups, grid points were classified according to three alternative hypotheses: for every grid point the estimated probability of belonging to a particular group was either significantly higher (*Hyp*_*1A*_), lower (*Hyp*_*1B*_) or no different to (*Hyp*_*0*_) the expectation from random spatial structure in the whole population estimated using bootstrap permutation. For the permutation, we randomly re-sampled the probabilities of belonging to different genetic groups, but did not change the placement of sample points. The confidence interval of the random distribution was estimated with a simple percentile method from the generated bootstrap distribution. Thus, according to the primary empirical values of each group, grid points could be classified as: 1) statistically significant core area of the group (corresponding to *Hyp*_*1A*_; within the upper 2.5% percentile of the bootstrap distribution); 2) probable range of the group (corresponding to *Hyp*_*0*_; between the 2.5% and 97.5% percentiles of the bootstrap distribution); or 3) evidently out of the range of the group (corresponding to *Hyp*_*1B*_; within the lower 2.5% percentile of the bootstrap distribution).

To identify potential regions of the study area that might represent corridors or barriers to migration, as well as core, transition and blending areas of population subgroups, we performed DResD analysis, which is a recently introduced spatially explicit, individual-based approach that is based on IBD modelling and pairwise geographic and genetic distances [7]. Genetic distance values were corrected for IBD, considering the spatial distances between pairs. The resulting residual values were interpolated throughout the study area using distance weighting (w = 1/dist.), based on the mid-locations of each sample pair (for a step-by-step guide to the DResD procedure, see Keis et al. [7]). We aimed to identify geographic regions where genetic distance between individuals is significantly higher or lower than is expected from the effect of IBD alone (the null-model), representing possible migration barriers or corridors, respectively. For genetic distance, we calculated the D_LR_ matrix with the DOH calculator, which takes genotypes of individuals from several populations and determines from which population each individual is most likely to have come. It uses the assignment index, the highest probability of an individual’s genotype in any of the populations [59]. Expected values at 5-km grid points were generated using 1000 randomisation iterations. As different mechanisms of gene flow have different spatial extents [60], the analysis was performed at three scales: using geographic distances between pairs of individuals 20-80 km apart (movements within the home range), 80-140 km apart (dispersal of juveniles and solitary individuals), and 140-250 km apart (large-scale migrations). For the DResD procedure applied in this study, see Information S1 for the full script in R 2.14 language [61].

### Genetic diversity and inbreeding

Software GENETIX [54] was used to estimate observed (*H*_*O*_) [62] and unbiased expected (*H*_*Eunb*_) heterozygosities [63], the number of alleles (*N*_*A*_) and inbreeding estimator Wright’s *F*_*IS*_ [64], for all samples and for each genetic group separately. Deviations from Hardy–Weinberg equilibrium were tested using GENEPOP v4.2. For each population–locus combination, departure from Hardy–Weinberg expectations was assessed using exact tests with unbiased P values estimated through a Markov chain method (set to 1000 batches of 10 000 iterations each and with 10 000 steps of dememorization); a global test across all loci and populations was performed using Fisher’s method [65]. We also tested for linkage disequilibrium between all pairs of loci in the Estonian-Latvian wolf population according to the method of Black and Kraftsur [66] implemented in GENETIX. FSTAT v.2.9.3 was used to calculate the allelic richness *A*_*R*_ [67], that would be obtained if sample sizes of all genetic groups were equal, using the rarefaction method of Petit et al. [68]. For rates of genetic differentiation and migrations between genetic groups, see Information S2.

### Effective population size

To estimate the effective population size of the Estonian-Latvian population, we used two methods that require only a single distinct genotypic population sample: (1) We estimated *Ne* (and 95% confidence limits) using the approximate Bayesian computation method implemented in the software ONESAMP 1.2 [69] with priors of 2 to 400 for *Ne*; (2) software LDNE v1.31 [70] was used to estimate the linkage disequilibrium based estimator of *Ne*. LDNE implements a recently developed bias correction [71] for estimates of effective population size. Since the social structure of the Estonian-Latvian wolf population may have been disrupted, we calculated a mean of monogamous and random mating, and excluded all alleles with frequencies less than 0.02.

## Results

### Genotyping error rates

None of the analysed 166 samples included more than one locus with missing alleles. The rate of allele dropouts was 0.004, the rate of false alleles 0.003 and the rate of other errors (double and complete errors) <0.001.

### Population bottlenecks

Allele frequency distributions revealed some evidence of recent population bottlenecks in the Estonian-Latvian wolf population. Allele frequencies had a typical L-shaped distribution (data not shown), indicating that no detectable shift in distribution had occurred and that the frequency of rare alleles had not dropped. In the sign test conducted on all 16 microsatellite loci, the signatures of bottleneck were detected with SMM and TPM models: wolf populations were not at mutation-drift equilibrium under SMM (P < 0.0001), with 16 loci out of 16 exhibiting heterozygosity deficiency; mutation-drift equilibrium was also not identified under TPM (P = 0.006; 12 loci with heterozygosity deficiency). Bottleneck was not statistically supported under the IAM model (P = 0.045).

### Genetic diversity and effective population size of Estonian-Latvian wolf population

For all 166 samples and 16 microsatellite loci, expected unbiased heterozygosity (*H*_*Eunb*_) was 0.73 and observed heterozygosity (*H*_*O*_) 0.75 (Table S1). The mean number of alleles per locus (*N*_*A*_) was 8.0 and the inbreeding coefficient was slightly negative (*F*_*IS*_ = -0.04). No significant linkage disequilibrium was found when all 166 samples were analysed together, but there was a statistically significant deviation from Hardy–Weinberg equilibrium, indicating heterozygosity deficiency.

### Detecting population structure

**Figure 2:**
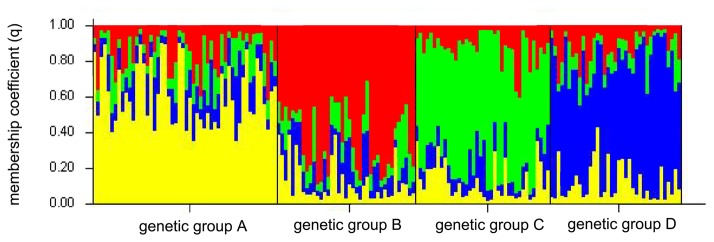
Bayesian admixture analysis of wolf genotypes from Estonia and Latvia, based on 16 autosomal microsatellite loci using Structure v2.2, *K* = 4 Each vertical bar represents the membership coefficient (*q*) for an individual wolf. A-D designate four genetic clusters.

**Figure 3:**
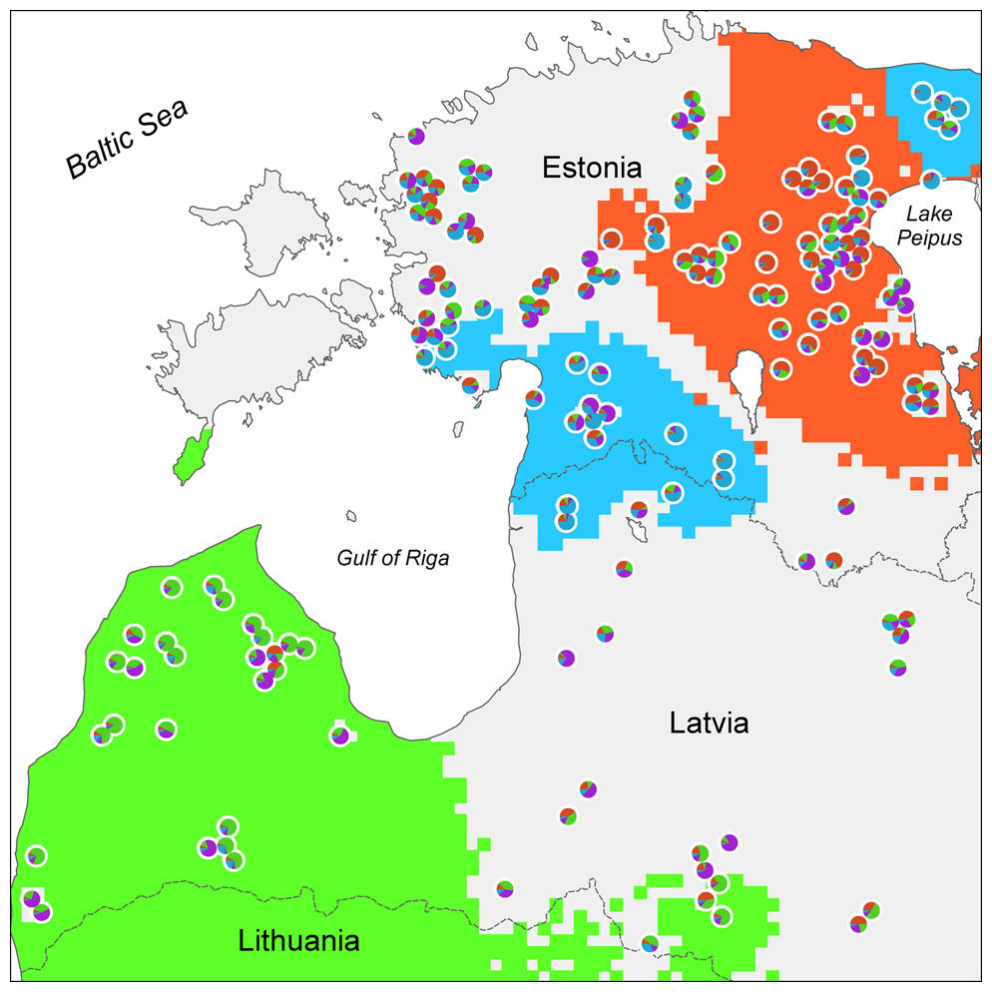
Geographical ranges of four genetic groups (A–D) in the Estonian-Latvian wolf population based on distance weighted interpolation of Structure membership coefficients. As determined by 1000 bootstrap permutations the dark coloured grid points (5×5 km) denote group core areas. Individuals are represented by multi-coloured pies which reflect the membership coefficient for each cluster (zoom to see the details).

**Figure 4:**
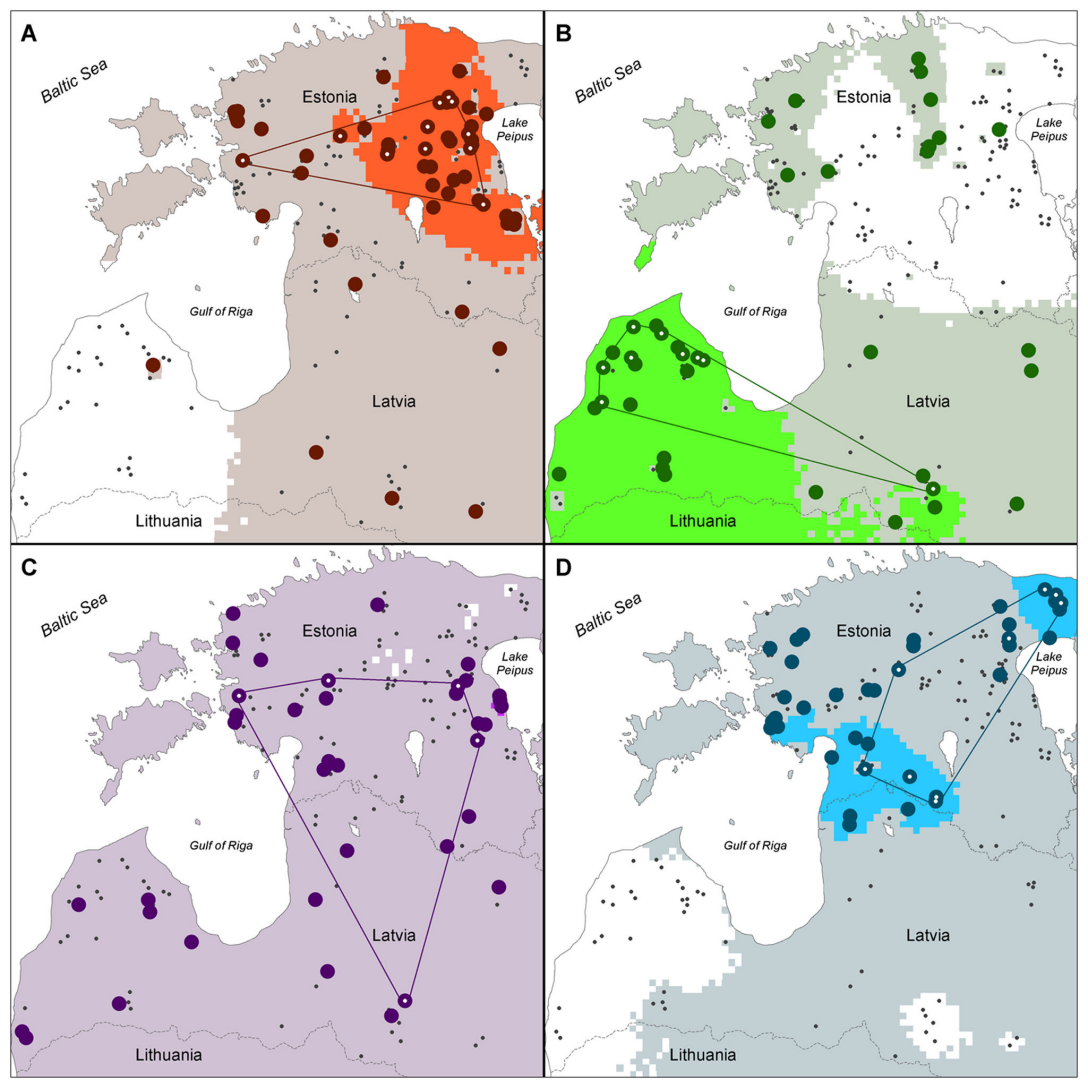
Geographical ranges of four genetic groups (A–D) presented separately. The dark coloured grid points (5×5 km) denote the core area of the group (as in Figure 3), whereas the light coloured areas represent near random group probability, and white areas are significantly outside of the range of the group.

**Figure 5:**
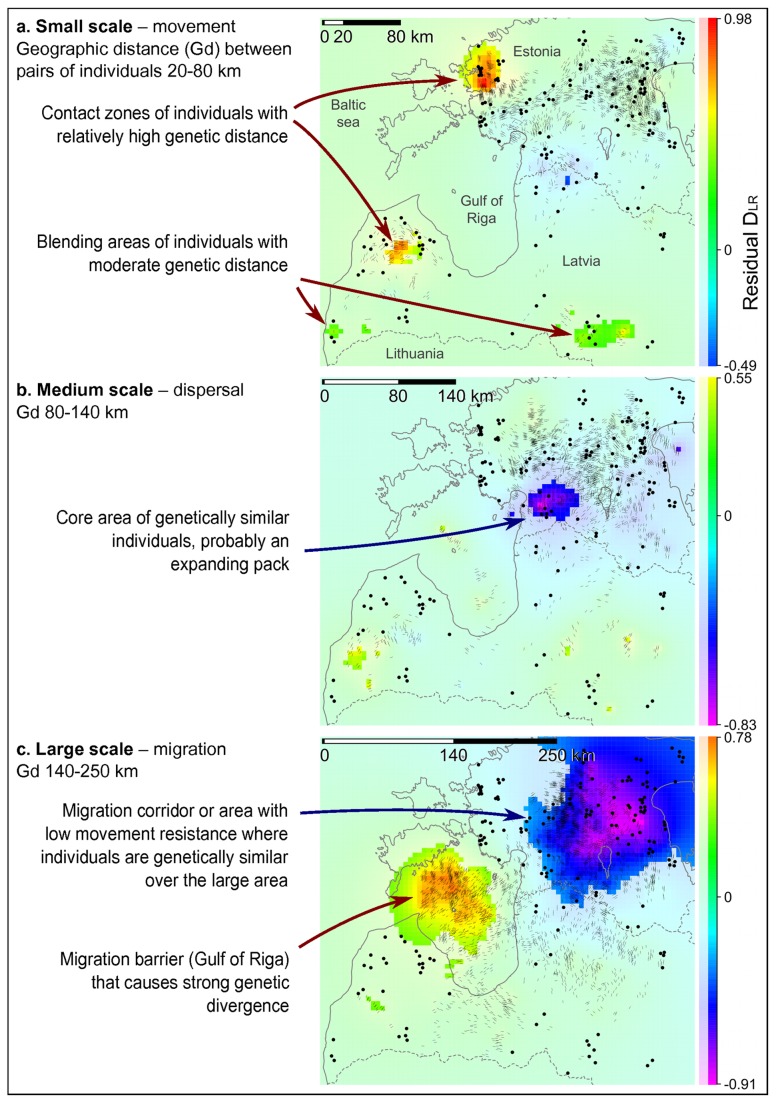
Spatial distribution of genetic differentiation between individuals in the Estonian-Latvian wolf population (n = 166) based on results of the spatially explicit DResD procedure at three spatial scales: (a-c) - the average D_LR_-index (based on 16 microsatellite loci) between sample pairs, corrected for isolation by distance and interpolated across the study area using inverse distance weighting. The full coloured areas represent the 5 km grid points where the tested value deviates significantly from the null-model (IBD alone – a value of 0; p ≤0.05 according to 1000 iterations). The dots represent sample locations, and dashes denote locations and directions of sample pair midpoints lying at a particular distance range; the black section of the scale-bar in the top-left corner of each image represents the distance range of sample pairs included in the respective calculation.

Cluster analysis using STRUCTURE and the method proposed by Evanno et al. [52] suggested the existence of four different genetic groups A-D (Figure 2, Figure S2). All genetic groups comprised individuals with a high average estimated membership coefficient for the respective group (Table 1). It is well known that interpreting STRUCTURE results may be challenging when IBD is present in the sampling set. Therefore, we estimated the effect of IBD and it turned out to be weak (R^2^ = 0.059; p < 0.001; Figure S3), explaining only 6% of the variation. Thus, as the effect of IBD was small, there were no incompatibilities with the assumptions of STRUCTURE. The structuring of the Estonian-Latvian wolf population into distinct genetic groups gained further support from FCA analysis (Figure S4) and from the linear interpolation approach, which clearly identified the geographical ranges of the groups (Figures 3, 4). According to the range of core (*Hyp*_*1A*_) grid points, three of the four genetic groups were geographically well defined: groups A (covering 12.3% of the analysed land area) and D (covering 7.9% in two separate core areas) were Estonian-based, whereas group B (26.3% coverage) was Latvian-based. However, group C was distributed throughout Estonia and Latvia, with almost all land area falling within the probable range (*Hyp*_*0*_) of the group (and a core area with only 0.1% coverage). The credible range of group B (*Hyp*_*1B*_ range) also included the majority of Estonia, while western Latvia was outside the credible ranges of groups B and D (Figures 3, 4). The DResD algorithm provided clear evidence of spatial variation of genetic divergence that is likely related to varying landscape resistance to individual movements. At each of the three spatial scales analysed, several areas appeared where the interpolated residual D_LR_ value was significantly higher or lower than expected from IBD alone (Figure 5). At the smallest spatial scale (20-80 km; Figure 5a) several blending areas of different groups appeared with relatively high genetic distance between otherwise geographically closely positioned individuals. At the medium scale (80-140 km) a putative territory of an expanding pack was detected in the forested area in south-west Estonia, coinciding with one of the core areas of group D (Figure 5b). At the largest spatial scale (140-250 km) a large area in the north-eastern part of Estonia was identified as a migration corridor, where individuals are genetically relatively similar over the large geographic distance. Moreover, the Gulf of Riga coincided with strong divergence between individuals (Figure 5c).

**Table 1:** The average estimated membership coefficients of individual Estonian and Latvian wolves in four genetic clusters.

Genetic group	Average probability of membership to clusters (*K* = 4)			
	1	2	3	4
A	**0.87**	0.08	0.12	0.07
B	0.03	**0.78**	0.11	0.06
C	0.04	0.07	**0.68**	0.05
D	0.03	0.07	0.09	**0.82**

The main cluster with the highest membership coefficient is in bold.

### Genetic diversity within genetic groups and effective population size

All 16 microsatellite loci were polymorphic in all genetic groups, with values of *H*_*Eunb*_ ranging from 0.51 to 0.88, and *H*_*O*_ ranging from 0.51 to 0.90, except for locus FH2017 in group D, where *H*_*Eunb*_ = 0.38 and *H*_*O*_ = 0.35. The mean number of alleles per locus (*N*_*A*_) ranged from 6.4-10.8 (Table S1). Wolves belonging to group C also exhibited more alleles per locus than any other group at 12 out of 16 loci. We found linkage disequilibrium between some pairs of loci in three of the four groups: after Bonferroni correction, there was (*P* < 0.005) linkage disequilibrium between five pairs of loci in group A, between one pair of loci in group B and between four pairs of loci in group C. The pairs of loci with significant linkage disequilibrium were different in all three groups. The inbreeding coefficient was negative in three of the four groups (A, C and D) and slightly positive in group B (Table S1). In groups A and D, both 95% bootstrapped (1000 permutations) confidence limits of the inbreeding coefficient were negative.

According to ONESAMP, the estimated mean effective population size in the whole sample set was 151.5 (95% CL = 140.9-166.2), and the corresponding estimate using LDNE was 138.0 (95% CL: parametric = 123.4-155.1; jack-knife = 125.2-159.8). For different genetic groups the estimated mean effective population size using the approximate Bayesian computation method was 61.8 individuals (95% CL = 54.5-91.5) for group A, 45.5 (95% CL = 41.4-53.1) for group B, 23.6 (95% CL = 21.6-28.4) for group C and 42.0 (95% CL = 37.4-53.3) for group D.

## Discussion

### Population bottlenecks and sub-structuring

The Estonian-Latvian wolf population is characterised by relatively high genetic diversity (see Table S1) despite past population bottlenecks and severe hunting pressure. The bottleneck signature was also detected in genetic data with program BOTTLENECK: it was statistically significant under the SMM and TPM models, but not under the IAM model. A possible explanation for this is that the SMM and TPM models fit better with the microsatellite mutation process in the wolf population under study. However, one of the assumptions for all these models is that no immigration and no population substructure exist. As these assumptions are to some extent violated in this study, it is difficult to have absolute certainty about the results of the bottleneck analysis, although it is known from demographic data that strong bottlenecks occurred around the mid-1960s.

Structuring of wild animal populations due to overexploitation and habitat degradation is of increasing conservation and management concern. Therefore, estimating genetic variation and the degree to which populations are genetically structured is important for conservation planning. The discovery of cryptic population structure in the Estonian and Latvian wolf population was unexpected due to the high mobility that the species exhibits, the relatively small geographic area studied, and the lack of obvious movement barriers and ecological specialisation. Nonetheless, analysis with STRUCTURE clearly identified four genetic groups in Estonia and Latvia (Figure 2, S2, and Table 1). A potential problem associated with identifying genetic groupings is the effect of IBD, which may produce artifactual partitions in weakly differentiated populations (e.g. [72]). However, we assessed the effect of IBD and found it to be negligible (Figure S3), suggesting that the genetic groups determined by STRUCTURE are realistic. Moreover, the splitting of group D (Figure 4d) into two geographically distant cores and the presence of group C (Figure 4c) throughout the whole study area are inconsistent with a pattern of structuring caused by IBD. In addition, we used spatial interpolation and detected several core areas of genetic groups where individuals with a high probability of belonging to other genetic groups were absent. This approach demonstrated that the core areas of three groups (A, B and D) had distinct geographic locations that did not overlap with each other (Figure 3). Areas significantly outside the genetic group ranges were also recorded (the white grid areas in Figure 4). To the best of our knowledge, this is the first study to use such an approach to statistically test spatial separation of genetic groups.

The factorial correspondence analysis with GENETIX [54] revealed three distinct and non-overlapping groups (B, C and A/D, i.e. it was not able to separate groups A and D from each other), suggesting that this approach had somewhat lover sensitivity (Figure S4). It has been proposed that with low genetic divergence between putative groups, kinship-based methods can facilitate the investigation of population structuring [73]. However, this approach requires almost complete sampling of target populations and was therefore not applicable in this study.

Several processes might be expected to have promoted the emergence of distinct genetic groups following a bottleneck in the study area: (1) groups might be formed by immigrant individuals from different parts of Lithuania and Russia; or (2) groups might originate from spatially separated local wolf packs (with no significant immigration).

Considering all the data, the most plausible scenario may be a combination of these two processes. We propose that the three groups with limited distribution and distinct cores (A, B and D) are likely to have arisen due to immigration of ‘foreign’ individuals, while the remaining widespread group (C) reflects a remnant population of local individuals.

After the severe population bottleneck in Estonia and Latvia in the mid-1960s, immigration from the wolf population in Russia could explain the geographic distribution of groups A and D, which are located near the Estonian-Russian border (Figures 3, 4). The dual core area of group D may have arisen during the post-bottleneck period, when immigrant wolves from Russia dispersed further towards south-west Estonia (note that wolves belonging to this group are visible along this putative migration axis) (Figure 4d), whereas severe and continuous hunting pressure in central and eastern Estonia during recent decades has fragmented this group into two geographically distant cores. Meanwhile, the distribution of groups A and B are consistent with immigration into Estonia from Pskov oblast, with subsequent expansion along a south-east north-west axis, and immigration from Lithuania, respectively.

The likely explanation for the homogeneous distribution of group C is that it is the most long-established group of the four, and that it was widespread in the study area before the severe population bottleneck in Estonia and Latvia in the mid-1960s. Due to severe hunting pressure, group C was probably present only at low density during the bottleneck, leaving vacant territories throughout its distribution area. During the population expansion period, vacant territories were probably recolonised both by group C wolves and immigrant wolves from other groups. Thus, according to our hypothesis, group C is the oldest and therefore the native group, whereas groups A, B and D have appeared during the post-bottleneck period due to immigration.

This of course has to be viewed as a relatively simple hypothesis. Given the interplay of hunting and immigration pressure, the detail of the real situation may be somewhat more complex. However, the question of how population sub-structuring has been maintained despite half a century elapsing since the bottleneck is perhaps more tractable. Differentiation between groups (*F*_*ST*_ = 0.04-0.07, see Table S2 and Information S2) is not large, reflecting their geographic proximity and a degree of migration between groups (Table S3). The wolf is one of the most mobile terrestrial mammal species, having the ability to disperse over long distances. Wabakken et al. [74] have documented a dispersal distance of 1,092 km from southeast Norway to northeast Finland. Thus, one might expect gene flow within Estonia and Latvia – a relatively limited geographic area (maximum extent 560 km) - to be considerable as there are no obvious movement barriers. Pilot et al. [75] have also demonstrated that wolf populations in Eastern Europe display a non-random spatial genetic structure in the absence of obvious physical barriers to movement, and suggested that ecological factors play an important role in population structuring. However, that study involved a much larger territory, and sub-structuring of the wolf population in Estonia and Latvia due to ecological factors is unlikely. Among other large carnivores, population sub-structuring has also been demonstrated in Europe for Eurasian lynx ( *Lynx* *lynx* ) [76] and for brown bear [6]; but in both cases, it occurred similarly at much large scales. The weak IBD effect observed in this study (Figure S3) suggests that wolves are capable of mixing all over the study area, suggesting that in the absence of hunting, observed groupings would probably merge. We propose that strong hunting pressure is the most likely the major factor that could maintain the observed population sub-structuring (see also below). In the Finnish wolf population, which has been subject only to mild hunting pressure (in 2000-2005 yearly about 6-10% of population), and inhabits a somewhat larger territory, no such sub-structuring has been recorded [77]. When social structure is intact, inbreeding is generally avoided and movement of individuals between packs does not allow fixation of pack-specific alleles and genetic differentiation into distinct groups [29,32].

### Is population sub-structuring affected by hunting?

Different methods for estimating population structure are based on the assumption that populations do not receive immigrants during the study interval (e.g. [51]). Thus, it is important to clarify whether the population structuring observed in this study is the result of severe hunting pressure or of significant recent immigration from neighbouring populations. Here we argue that wolf-hunting is the primary cause.

First of all, even if significant immigration exists, it is highly likely to be a consequence of hunting, since this has without doubt been the primary mechanism responsible for reducing the density of the Estonian-Latvian wolf population. In established wolf populations with no hunting (or with low hunting pressure) population density is limited by the carrying capacity of the ecosystem, and under these circumstances, immigration rates remain low [30]. However, under severe hunting pressure, immigration rates are highly likely to increase due to appearance of vacant territories. Thus, even if immigration explains a degree of population structure, hunting, through its effect on immigration rates, almost certainly remains the ultimate cause.

**Figure 6:**
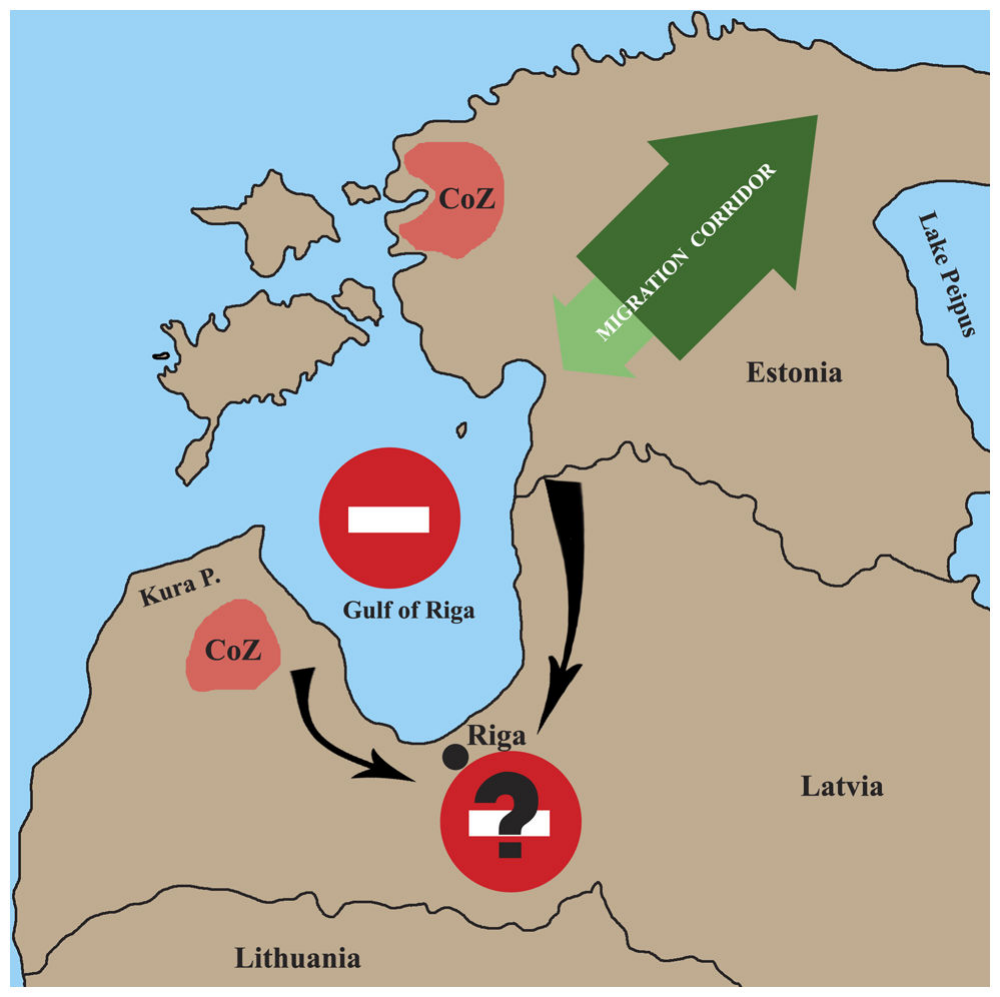
Schematic representation of DResD results (shown in Figure 5). Migration directions and strength were determined with BayesAss (see Information S2), with dark green arrows indicating stronger and light green arrow weaker dispersal strength. Red prohibiting signs designate migration barriers (note that the barrier in the form of the Gulf of Riga was clearly identified by the analysis, whereas the city of Riga and its surrounding infrastructure are proposed to explain the evidence for a barrier in that approximate location). CoZ: contact zones for genetically distant individuals.

Besides this, there are further reasons to believe that hunting is a more important direct cause than immigration of the maintenance of population sub-structuring. In Finland [77,78,79] and Lithuania [80], where hunting pressure is low and immigration moderate, no population structuring has been observed. Therefore, on the basis of immigration alone, the much smaller territory of Estonia and Latvia might be expected to exhibit no population structuring at all. If significant immigration were ongoing, one might expect to detect genetic groups near the border areas with Lithuania and Russia. However, we did not detect such groups or even rare alleles (data not shown) near the borders of our study areas. In group D, the higher migration rate from the south-western core area towards the north-east supports the idea that immigration from Russia has recently been small (Figure S5, Figure 6). Moreover, one would expect to see (with the DResD analysis) clustering of individuals with relatively high genetic distance near border areas if recent immigration were significant. However, such areas were only detected far from the border areas (Figures 5, 6). The only indication that a low level of recent immigration may have occurred comes from the blending areas of individuals with moderate genetic distance in the southern and eastern parts of Latvia near border with Lithuania (Figure 5a). Indeed, small scale immigration from neighbouring areas is likely to occur, especially to Latvia, as there is no evident movement barrier. However, this pattern could have also arisen due to hunting-driven migration processes inside the study area, and it is unlikely that the current, and most probably low, immigration rate is capable of maintaining the observed population sub-structure.

### The Interpolation and DResD methods

We propose an iterative linear interpolation method incorporating bootstrap permutations to investigate whether genetic groups are spatially distinct. The method allows users to further investigate STRUCTURE output in order to reveal both core areas and areas of low significance for genetic groups, and, as such, represents a novel spatial genetic approach. Here we used this approach to demonstrate that three of the detected genetic groups have geographically non-overlapping core areas (Figure 3).

The DResD procedure represents a recently developed spatially explicit, individual-based approach for identifying migration corridors and barriers [7]. It is a tool for the analysis of genetic data in a geographical context that is applicable to any data that yield genetic distance matrices, including sequence data, microsatellites, amplified fragment length polymorphisms (AFLPs) and single nucleotide polymorphisms (SNPs). We consider the DResD procedure to provide several benefits in landscape genetics analysis: it uses an individual based geographically explicit approach, treats population genetic composition as a continuous spatial variable, and accounts for the effect of IBD in calculations. This study is the first time it has been used for analysis of microsatellite data and it provides good evidence of the ability of the DResD procedure to detect transition and contact zones, and to reveal areas with high and low movement resistance (migration corridors and barriers) at different spatial scales (Figures 5, 6). At the small scale, contact areas for genetically distant individuals indicated areas where territories of wolves from different genetic groups are present (Figure 5a). At the medium geographic scale, an area containing closely related individuals was found that most likely represented the range of an expanding pack (Figure 5b). At the largest scale, analysis revealed a putative migration area and identified the Gulf of Riga as an efficient movement barrier (Figure 5c). Wolves could hypothetically move around the gulf, but the analysis shows that they do not, suggesting that the city of Riga and its surrounding infrastructure act also as a barrier (Figure 6). Used in conjunction with the other analyses, including the interpolation approach, it provides important details about population sub-structure and population processes.

## Supporting Information

Table S1 **Genetic diversity data for gray wolf genetic groups A-D in the Estonian-Latvian wolf population.** Number of alleles (N_A_), allelic richness independent of sample size (A_R_) (using Fstat) allelic richness estimated by rarefaction and based on a minimum sample size n = 37; expected unbiased heterozygosity (H_Eunb_) and observed heterozygosity (H_O_) and inbreeding estimator Wright’s *F*_*IS*_. 95% CLs for mean *F*_*IS*_ are shown in parentheses. **P < 0.01; *P < 0.05.
(DOCX)

Table S2 **Comparisons of pairwise *F*_*ST*_ values for genetic groups A-D in the Estonian-Latvian wolf population.**
(DOCX)

Table S3 **Migration rates between genetic groups A-D in the Estonian-Latvian wolf population based on the results of software Bayesass v1.3.**
(DOCX)

Figure S1 **Number of wolves counted and hunted in Estonia and Latvia during 1995-2011.** Data from Estonian and Latvian official hunting statistics (based on assessment by local hunters).
(EPS)

Figure S2 **Rate of change in log-likelihood values (ΔK) for the number of clusters estimated by Structure v2.2.** (The maximal value of ΔK indicates the most likely number of clusters.)
(TIF)

Figure S3 **Isolation by distance (IBD).** Dependence of pairwise genotype likelihood ratio distance (D_LR_) on geographic distance in the Estonian-Latvian wolf population based on 166 samples (13 695 pairs). The reverse exponential asymptotic fit represents the curve of IBD. Nonparametric Mantel test: R^2^ = 0.059, p < 0.001.
(TIF)

Figure S4 **Factorial correspondence analysis (Genetix) of wolves belonging to four genetic groups (A–D) in Estonia and Latvia.** The analysis is based on 16 microsatellite loci (see Figure S1).
(TIF)

Figure S5 **Migration rates among the four genetic groups A-D in the Estonian-Latvian wolf population.** Based on the results of software Bayesass v1.3 (thicker arrows denote higher migration rates).
(TIF)

Information S1 **The R 2.14 code used in the DResD procedure.**
(DOC)

Information S2 **Genetic differentiation and migrations between four genetic groups.**
(DOC)
