# Supplementary material for: Spatial Genetic Analyses Reveal Cryptic Population Structure and Migration Patterns in a Continuously Harvested Grey Wolf (Canis lupus) Population in North-Eastern Europe
Source: PLoS One. 2013 Sep 19;8(9):e75765. doi: 10.1371/journal.pone.0075765 (PMC3777892; doi:10.1371/journal.pone.0075765)
Supplement: Table S1 — Genetic diversity data for gray wolf genetic groups A-D in the Estonian-Latvian wolf population. Number of alleles (NA), allelic richness independent of sample size (AR) (using Fstat) allelic richness estimated by rarefaction and based on a minimum sample size n = 37; expected unbiased heterozygosity (HEunb) and observed heterozygosity (HO) and inbreeding estimator Wright’s F IS. 95% CLs for mean F IS are shown in parentheses. **P < 0.01; *P < 0.05. (DOCX) [file pone.0075765.s001.docx]

**Table S1.** **Genetic diversity data for gray wolf genetic groups A-D in the Estonian-Latvian wolf population.** Number of alleles (*N_A_*), allelic richness independent of sample size (*A_R_*) (using Fstat) allelic richness estimated by rarefaction and based on a minimum sample size n = 37; expected unbiased heterozygosity (*H_Eunb_*) and observed heterozygosity (*H_O_*) and inbreeding estimator Wright’s *F_IS_*. 95% CLs for mean *F_IS_* are shown in parentheses. ***P* < 0.01; **P* < 0.05

| Locus | Group A (N=52) | | | | | Group B (N=39) | | | | | Group C (N =38) | | | | | Group D (N=37) | | | | |
| --- | --- | --- | --- | --- | --- | --- | --- | --- | --- | --- | --- | --- | --- | --- | --- | --- | --- | --- | --- | --- |
|  | *N_A_* | *A_R36_* | *H_Eunb_* | *H_o_* | *F_IS_* | *N_A_* | *A_R36_* | *H_Eunb_* | *H_o_* | *F_IS_* | *N_A_* | *A_R36_* | *H_Eunb_* | *H_o_* | *F_IS_* | *N_A_* | *A_R36_* | *H_Eunb_* | *H_o_* | *F_IS_* |
| FH2001 | 9 | 8.257 | 0.776 | 0.885 | -0.151* | 6 | 5.999 | 0.690 | 0.718 | -0.054 | 12 | 11.84 | 0.861 | 0.816 | 0.040* | 9 | 8.892 | 0.799 | 0.757 | 0.040 |
| FH2010 | 5 | 4.692 | 0.696 | 0.808 | -0.172 | 6 | 5.923 | 0.763 | 0.718 | 0.047 | 16 | 15.58 | 0.858 | 0.816 | 0.037* | 4 | 4 | 0.674 | 0.676 | -0.016 |
| FH2017 | 4 | 3.998 | 0.512 | 0.538 | -0.062 | 6 | 5.846 | 0.643 | 0.615 | 0.030 | 13 | 12.74 | 0.773 | 0.789 | -0.034* | 4 | 3.973 | 0.382 | 0.351 | 0.069 |
| FH2054 | 11 | 9.584 | 0.812 | 0.750 | 0.068 | 9 | 8.841 | 0.785 | 0.692 | -0.107 | 16 | 15.68 | 0.799 | 0.842 | -0.069 | 6 | 5.973 | 0.643 | 0.784 | -0.235 |
| FH2079 | 8 | 7.05 | 0.748 | 0.865 | -0.169 | 7 | 6.841 | 0.647 | 0.692 | -0.084 | 17 | 16.73 | 0.875 | 0.842 | 0.025* | 6 | 5.919 | 0.572 | 0.676 | -0.198 |
| FH2088 | 6 | 5.916 | 0.782 | 0.843 | -0.089 | 7 | 6.913 | 0.788 | 0.667 | 0.143* | 9 | 8.893 | 0.810 | 0.842 | -0.054 | 6 | 5.973 | 0.611 | 0.676 | -0.121 |
| FH2096 | 6 | 5.684 | 0.688 | 0.712 | -0.045 | 6 | 5.918 | 0.717 | 0.590 | 0.166* | 7 | 6.943 | 0.740 | 0.789 | -0.081 | 6 | 5.999 | 0.747 | 0.649 | 0.119* |
| C466 | 8 | 6.761 | 0.637 | 0.731 | -0.158 | 7 | 6.846 | 0.796 | 0.795 | -0.012 | 11 | 10.84 | 0.721 | 0.684 | 0.038* | 6 | 6 | 0.659 | 0.865 | -0.330* |
| vWF | 8 | 7.388 | 0.758 | 0.608 | 0.190* | 9 | 8.836 | 0.766 | 0.513 | 0.321* | 12 | 11.84 | 0.805 | 0.789 | 0.006 | 4 | 3.973 | 0.539 | 0.703 | -0.322* |
| AHT130 | 12 | 10.57 | 0.780 | 0.846 | -0.095 | 7 | 6.998 | 0.747 | 0.763 | -0.035 | 9 | 8.943 | 0.814 | 0.789 | 0.017* | 8 | 7.973 | 0.800 | 0.784 | 0.006* |
| C09.173 | 7 | 6.377 | 0.751 | 0.731 | 0.018 | 11 | 10.62 | 0.791 | 0.872 | -0.117* | 8 | 7.973 | 0.826 | 0.865 | -0.061 | 10 | 10 | 0.856 | 0.833 | 0.013 |
| C20.253 | 7 | 6.684 | 0.792 | 0.827 | -0.054 | 9 | 8.841 | 0.821 | 0.821 | -0.013 | 9 | 8.895 | 0.786 | 0.789 | -0.018 | 7 | 6.973 | 0.672 | 0.757 | -0.142 |
| CXX22 | 5 | 4.6 | 0.669 | 0.654 | 0.013* | 5 | 4.995 | 0.696 | 0.897 | -0.306* | 8 | 7.943 | 0.718 | 0.842 | -0.189 | 4 | 3.973 | 0.588 | 0.676 | -0.166 |
| MCPH2 | 6 | 5.972 | 0.726 | 0.846 | -0.177 | 6 | 5.923 | 0.778 | 0.718 | 0.065 | 7 | 6.895 | 0.763 | 0.816 | -0.084 | 7 | 6.946 | 0.673 | 0.730 | -0.099 |
| MCPH4 | 8 | 7.564 | 0.710 | 0.788 | -0.121 | 8 | 7.841 | 0.759 | 0.718 | 0.042 | 8 | 8 | 0.842 | 0.763 | 0.082* | 7 | 6.946 | 0.710 | 0.730 | -0.042 |
| MCPH12 | 12 | 10.98 | 0.686 | 0.788 | -0.160 | 8 | 7.99 | 0.814 | 0.897 | -0.117 | 11 | 10.79 | 0.689 | 0.789 | -0.161 | 8 | 7.919 | 0.539 | 0.568 | -0.067 |
| ***Mean*** | ***7.625*** | ***7.005*** | ***0.720*** | ***0.764*** | ***- 0.07***  ***(– 0.098; -0.04)*** | ***7.313*** | ***7.198*** | ***0.750*** | ***0.730*** | ***0.012***  ***(– 0.041; 0.026)*** | ***10.813*** | ***10.656*** | ***0.793*** | ***0.804*** | ***-0.032***  ***(– 0.065; 0.031)*** | ***6.375*** | ***6.339*** | ***0.654*** | ***0.701*** | ***-0.093***  ***(– 0.156; – 0.062)*** |
