# Supplementary material for: Spatial Genetic Analyses Reveal Cryptic Population Structure and Migration Patterns in a Continuously Harvested Grey Wolf (Canis lupus) Population in North-Eastern Europe
Source: PLoS One. 2013 Sep 19;8(9):e75765. doi: 10.1371/journal.pone.0075765 (PMC3777892; doi:10.1371/journal.pone.0075765)
Supplement: Table S2 — Comparisons of pairwise FST values for genetic groups A-D in the Estonian-Latvian wolf population. (DOCX) [file pone.0075765.s002.docx]

**Table S2.** **Comparisons of pairwise *F_ST_* values above the diagonal and pairwise values of derived estimates of *Nm* below the diagonal for genetic groups A-D in the Estonian-Latvian wolf population.** In comparisons of pairwise *F_ST_* *P < 0.05, number of permutations = 1023.

| Genetic group | A | B | C | D |
| --- | --- | --- | --- | --- |
| A | - | 0.05* | 0.04* | 0.05* |
| B | 4.97 | - | 0.04* | 0.07* |
| C | 7.07 | 6.25 | - | 0.05* |
| D | 5.33 | 3.53 | 4.66 | - |
