# Supplementary material for: Spatial Genetic Analyses Reveal Cryptic Population Structure and Migration Patterns in a Continuously Harvested Grey Wolf (Canis lupus) Population in North-Eastern Europe
Source: PLoS One. 2013 Sep 19;8(9):e75765. doi: 10.1371/journal.pone.0075765 (PMC3777892; doi:10.1371/journal.pone.0075765)
Supplement: Table S3 — Migration rates between genetic groups A-D in the Estonian-Latvian wolf population based on the results of software Bayesass v1.3. (DOCX) [file pone.0075765.s003.docx]

**Table S3.** **Migration rates between genetic groups A-D in the Estonian-Latvian wolf population based on the results of software Bayesass v1.3.** Means of the posterior distributions of *m,* the migration rate into each genetic group, are shown. 95% confidence limits are shown in parentheses. The groups into which individuals migrated are listed in the rows, while the origins of the migrants are listed in the columns. Values along the diagonal are the proportions of individuals derived from the source genetic group each generation. Migration rates ≥ 0.10 are in bold.

| Genetic group | Group A | Group B | Group C | Group D |
| --- | --- | --- | --- | --- |
| Group A | **0.69** (0.67; 0.72) | 0.00 (0.00; 0.02) | 0.00 (0.00; 0.02) | 0.30 (0.27; 0.32) |
| Group B | 0.02 (0.00; 0.06) | **0.84** (0.77; 0.95) | 0.00 (0.00^;^ 0.02) | 0.13 (0.02; 0.21) |
| Group C | 0.02 (0.00; 0.05) | 0.06 (0.02; 0.13) | **0.71** (0.68; 0.77) | 0.21 (0.13; 0.27) |
| Group D | 0.00 (0.00; 0.02) | 0.00 (0.00^;^ 0.02) | 0.00 (0.00; 0.02) | **0.96** (0.94; 0.99) |
