# Supplementary figures and images for: Spatial Genetic Analyses Reveal Cryptic Population Structure and Migration Patterns in a Continuously Harvested Grey Wolf (Canis lupus) Population in North-Eastern Europe
Source: PLoS One. 2013 Sep 19;8(9):e75765. doi: 10.1371/journal.pone.0075765 (PMC3777892; doi:10.1371/journal.pone.0075765)

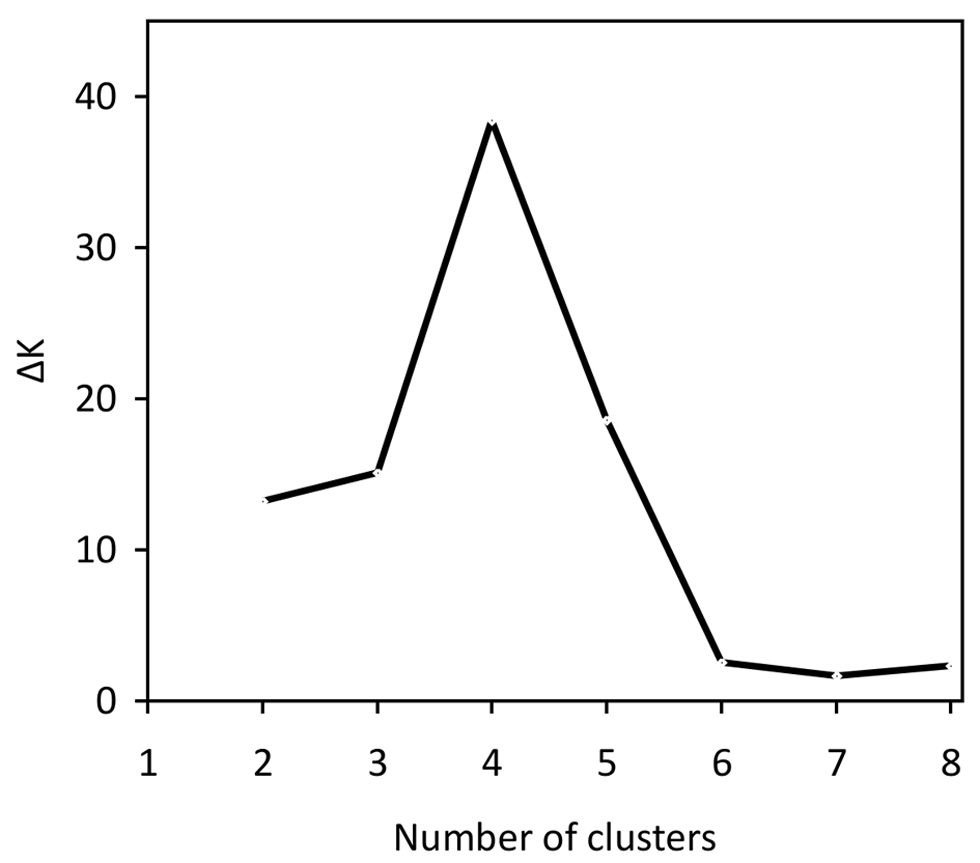

Supplement: Figure S2 — Rate of change in log-likelihood values (ΔK) for the number of clusters estimated by Structure v2.2. (The maximal value of ΔK indicates the most likely number of clusters.) (TIF) [file pone.0075765.s005.tif]

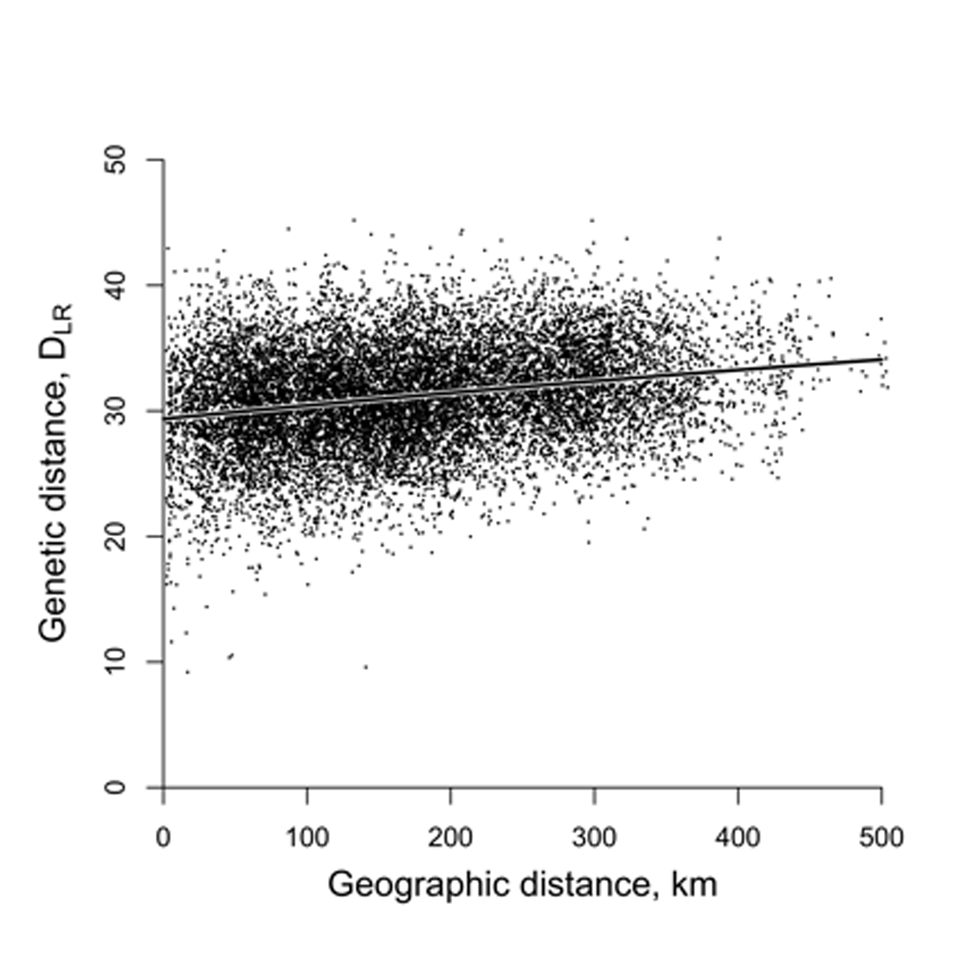

Supplement: Figure S3 — Isolation by distance (IBD). Dependence of pairwise genotype likelihood ratio distance (DLR) on geographic distance in the Estonian-Latvian wolf population based on 166 samples (13 695 pairs). The reverse exponential asymptotic fit represents the curve of IBD. Nonparametric Mantel test: R2 = 0.059, p < 0.001. (TIF) [file pone.0075765.s006.tif]

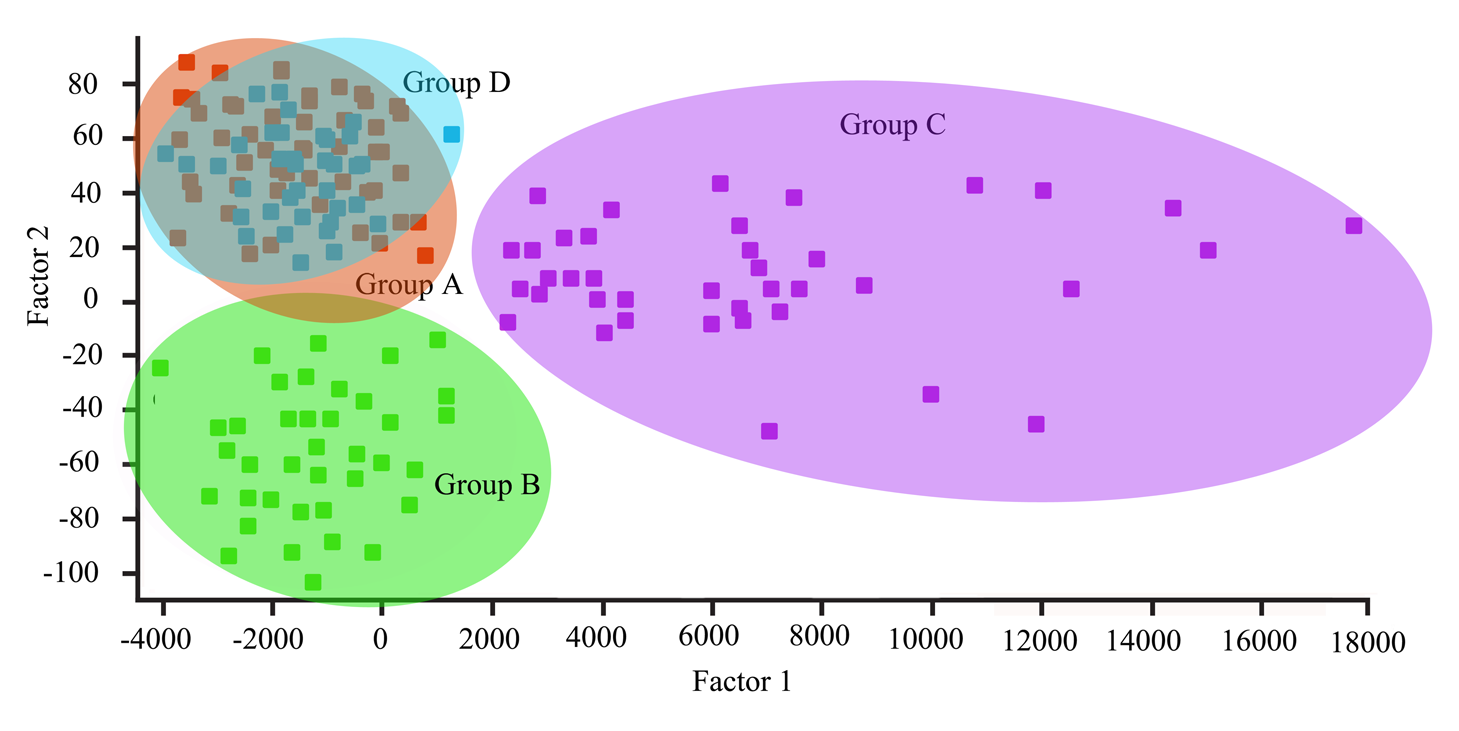

Supplement: Figure S4 — Factorial correspondence analysis (Genetix) of wolves belonging to four genetic groups (A–D) in Estonia and Latvia. The analysis is based on 16 microsatellite loci (see Figure S1). (TIF) [file pone.0075765.s007.tif]

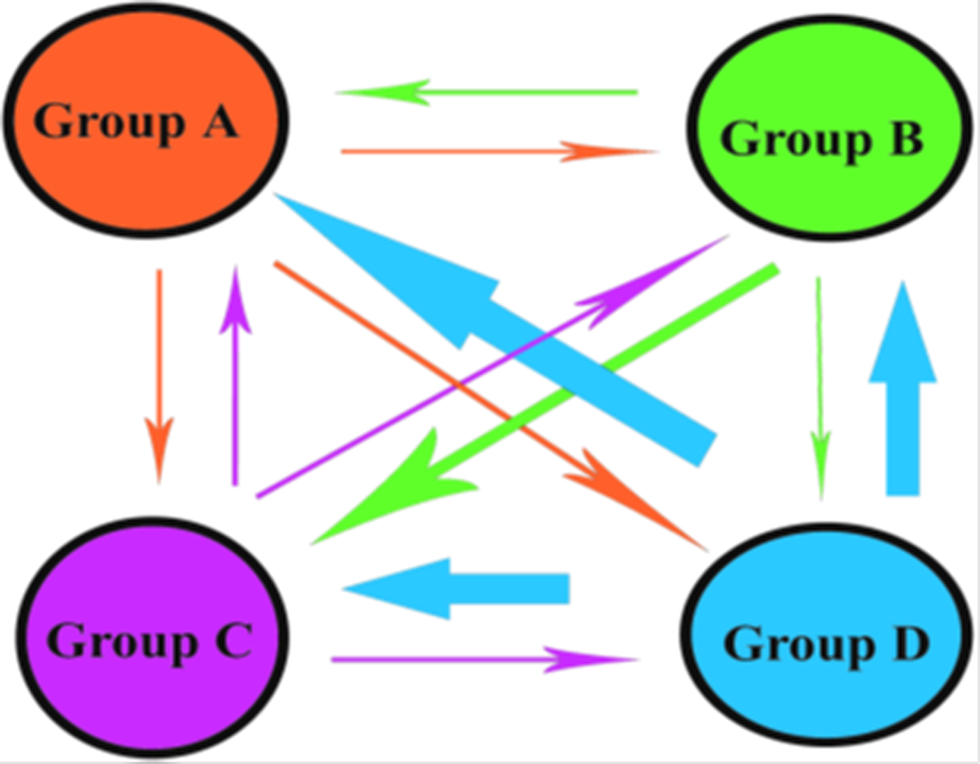

Supplement: Figure S5 — Migration rates among the four genetic groups A-D in the Estonian-Latvian wolf population. Based on the results of software Bayesass v1.3 (thicker arrows denote higher migration rates). (TIF) [file pone.0075765.s008.tif]
