# Supplementary material for: Spatial Genetic Analyses Reveal Cryptic Population Structure and Migration Patterns in a Continuously Harvested Grey Wolf (Canis lupus) Population in North-Eastern Europe
Source: PLoS One. 2013 Sep 19;8(9):e75765. doi: 10.1371/journal.pone.0075765 (PMC3777892; doi:10.1371/journal.pone.0075765)
Supplement: Information S1 — The R 2.14 code used in the DResD procedure. (DOC) [file pone.0075765.s009.doc]

**Information S1**. **The DResD procedure code in R 2.14 language with the parameter values used.** For detailed explanation of the method see Keis *et al*. 2013, “Complete mitochondrial genomes and a novel spatial genetic method reveal cryptic phylogeographic structure and migration patterns among brown bears in north-western Eurasia.” Journal of Biogeography, 40: 915–927). For additional discussion please contact Jaanus Remm (jaanus.remm@ut.ee).

**'Load and prepare data from tab-delimited TXT files'**

M.gen.diss <- read.table('*file path*', sep = '\t', header = T, row.names = 1, fill=T)

M.gen.diss <- as.dist(M.gen.diss)

sample.points <- read.table('*file path*', sep = '\t', header = T, row.names = 1, fill = T)

M.geo.dist <- dist(sample.points[,1:2])

IBD.data <- data.frame(cbind(as.vector(M.geo.dist), as.vector(M.gen.diss)))

**'Predict IBD-curve and calculate residual values of genetic distance'**

IBD.model.prelim <- NLSstAsymptotic(sortedXyData(x = IBD.data[1:250,1], y = IBD.data[1:250,2]))

IBD.model <- nls(formula = gen.diss ~ b0 + b1 * (1 - exp(-exp(lrc) * geo.dist)), data = IBD.data, start = list(b0 = IBD.model.prelim [1], b1 = IBD.model.prelim [2], lrc = IBD.model.prelim [3]))

M.IBD.res <- M.gen.diss - (coef(IBD.model)[1] + coef(IBD.model)[2] * (1 - exp(-exp(coef(IBD.model)[3]) * M.geo.dist)))

**'Grid parameters'**

step <- 5000

margin <- 10000

x.min <- min(sample.points[,1]) - margin

x.max <- max(sample.points[,1]) + margin

y.min <- min(sample.points[,2]) - margin

y.max <- max(sample.points[,2]) + margin

**'Midpoint parameters'**

n <- dim(sample.points)[1]

M.x1 <- as.dist(matrix(data = sample.points[,1], nrow = n, ncol = n, byrow = F))

M.y1 <- as.dist(matrix(data = sample.points[,2], nrow = n, ncol = n, byrow = F))

M.x2 <- as.dist(matrix(data = sample.points[,1], nrow = n, ncol = n, byrow = T))

M.y2 <- as.dist(matrix(data = sample.points[,2], nrow = n, ncol = n, byrow = T))

M.mid.x <- (M.x1 + M.x2) / 2

M.mid.y <- (M.y1 + M.y2) / 2

M.mid.azim <- atan((M.x2 - M.x1) / (M.y2 - M.y1)) * (180 / pi)

**'Deffinition of funcrions weighted.var, calculation and perm.rowscols'**

weighted.var <- function(x, w, na.rm = F){

if (na.rm){

w <- w[i <- !is.na(x)]

x <- x[i]

}

sum.w <- sum(w)

sum.w2 <- sum(w ** 2)

mean.w <- sum(x * w) / sum(w)

(sum.w / (sum.w ** 2 - sum.w2)) * sum(w * (x - mean.w) ** 2, na.rm = na.rm)

}

calculation <- function(gridx, gridy, midpoints, shift){

w <- 1 / (shift + sqrt((gridx - midpoints[,1]) ** 2 + (gridy - midpoints[,2]) ** 2))

return(c(weighted.mean(midpoints[,3], w), weighted.var(midpoints[,3], w) ** 0.5))

}

perm.rowscols = function (m) {

d <- dim(m)

s <- sample(1:d[1])

m[s,s]

}

**'Generate grid, initiate a table for middashes, and define cutoff values of three spatial scales'**

grid <- expand.grid(x = seq(from = x.min, to = x.max, by = step), y = seq(from = y.min, to = y.max, by = step))

middashes <- data.frame(numeric(0), numeric(0), numeric(0), numeric(0), numeric(0))

dist.cutoffs <- c(20000, 80000, 140000, 250000)

**'A routine that repeat the DResD calculations three times and save results into a table'**

for (i in 1:(length(dist.cutoffs) - 1)) {

min.dist <- dist.cutoffs[i]

max.dist <- dist.cutoffs[i+1]

shift <- 2500

midpoints <- data.frame(as.vector(M.mid.x[(M.geo.dist > min.dist) & (M.geo.dist < max.dist)]), as.vector(M.mid.y[(M.geo.dist > min.dist) & (M.geo.dist < max.dist)]), as.vector(M.IBD.res[(M.geo.dist > min.dist) & (M.geo.dist < max.dist)]), as.vector(M.mid.azim[(M.geo.dist > min.dist) & (M.geo.dist < max.dist)]))

middashes <- rbind(middashes, data.frame(i=i, x1=midpoints[,1] - 1500 * sin(midpoints[,4]/(180/pi)), y1=midpoints[,2] - 1500 * cos(midpoints[,4]/(180/pi)), x2=midpoints[,1] + 1500 * sin(midpoints[,4]/(180/pi)), y2=midpoints[,2] + 1500 * cos(midpoints[,4]/(180/pi))))

grid <- cbind(grid, t(mapply(calculation, grid[,1], grid[,2], MoreArgs = list(midpoints, shift))), 0, NA)

n.resamp <- 1000

for(j in 1:n.resamp) {

M.IBD.res.resamp <- as.dist(perm.rowscols(as.matrix(M.IBD.res)))

midpoints.resamp <- data.frame(midpoints[,1:2], as.vector(M.IBD.res.resamp[(M.geo.dist > min.dist) & (M.geo.dist < max.dist)]))

resampled <- t(mapply(calculation, grid[,1], grid[,2], MoreArgs = list(midpoints.resamp, step)))

grid[,5+(i-1)*4] <- grid[,5+(i-1)*4] + (grid[,3+(i-1)*4] < resampled[,1]) / n.resamp

next

}

grid[,6+(i-1)*4][grid[,5+(i-1)*4] > 0.025 & grid[,5+(i-1)*4] < 0.975] <- 1

next

}

**'Save results'**

names(grid) <- c('x', 'y', 'z1', 'SD1', 'p.bts>z1', 'insignif1', 'z2', 'SD2', 'p.bts>z2', 'insignif2', 'z3', 'SD3', 'p.bts>z3', 'insignif3')

names(middashes) <- c('i','x1','y1','x2', 'y2')

write.table(grid, '*file path*')

write.table(middashes, '*file path*')
