# Supplementary material for: Spatial Genetic Analyses Reveal Cryptic Population Structure and Migration Patterns in a Continuously Harvested Grey Wolf (Canis lupus) Population in North-Eastern Europe
Source: PLoS One. 2013 Sep 19;8(9):e75765. doi: 10.1371/journal.pone.0075765 (PMC3777892; doi:10.1371/journal.pone.0075765)
Supplement: Information S2 — Genetic differentiation and migrations between four genetic groups. (DOC) [file pone.0075765.s010.doc]

**Information S2. Genetic diversity, genetic differentiation and migrations between four genetic groups of Estonian-Latvian wolf population**

FStat v.2.9.3 was used to calculate the pairwise values of *F_ST_* [1]. The average number of migrants (*Nm*) was identified using Slatkin´s [2] private allele method in Genepop v. 4.1.4 [3]. We also used a Bayesian method developed by Wilson and Rannala [4] for estimating migration rates and to calculate the proportion of migrants (*m*) between different genetic groups. For this, the dataset was analysed using Bayesass v.1.3 [4] for 3 × 10^6^ iterations, with the first 1 × 10^6^ iterations omitted and samples taken after every 2000 iterations. The analysis was run three times and the values were averaged. In the first run, default settings were used, but the subsequent runs included different delta values.

Pairwise estimates of *Φ_ST_* and migration rates *Nm* between groups (Table S2) suggested least genetic differentiation between groups A and C. The genetic composition of the Latvian-based group (B) was more similar to group C than to the two Estonian-based groups, A and D. Indeed, the largest *Φ_ST_* values and lowest gene flow were observed between the groups B and D. Overall, the *Φ_ST_* –based method estimated *Nm* = 2.5 as the average effective number of migrants exchanged per generation between the genetic groups. The private allele method [2] provided an approximately 2.5-times higher estimate of the number of migrants: the mean frequency of private alleles among the groups was 0.02, and based on this frequency an overall estimate of *Nm* = 6.3 between all groups was produced. Pairwise estimates suggested that the mean number of wolf migrants per generation would be 6.9 between groups A and C and 3.3 between groups B and D. The Bayesian approach to estimate asymmetrical migration rates between the groups suggested that self-recruitment was high in group D (*m* = 0.96) but lower in groups A (*m* = 0.69), B (*m* = 0.84) and C (*m* = 0.71) (Table S3, Fig. S5). The results reflect asymmetric migration rates between the groups. Also, the estimates of migration rates from group D to all other groups were relatively high: *m* = 0.13-0.30; while group C received migrants from groups A (*m* = 0.02) and B (*m* = 0.06) besides group D (Table S3). We also estimated the migration rates inside group D, between two cores areas. The migration from the north-eastern core area to the south-western was 0.19 (*sd* = 0.03) whereas the migration rate in the opposite direction was 0.29 (*sd* = 0.13).

From the indirect approaches that use population differentiation to estimate the amount of migration between groups, the private allele approach gave the higher estimate (6.3) and the *Φ_ST_* -based method a lower estimate (3.3) for the number of migrants between the four genetic groups per generation. The Bayesian approach to estimate asymmetrical migration suggested that the movement of animals between groups is predominantly unidirectional. For example, group C received a substantially higher number of migrants from all other groups than it contributes (Table S3, Fig. S5). However, the results need to be interpreted with a degree of caution as Bayesass performs best at high F_ST_ values between groups. Asymmetric migration rates have also been observed in other wolf populations. The migration rate from the Karelian to the Finnish population was lower than in the opposite direction [5]. Similarly, studies of North American wolf populations [6,4,7,8] have shown that most studied populations have asymmetrical migration rates.

**References**

1. Weir BS, Cockerham CC (1984) Estimating F-statistics for the analysis of population structure. Evolution, 38: 1358-1370.

2. Slatkin M (1985) Rare alleles as indicators of gene flow. International Journal of Organic Evolution, 39: 53–65.

3. Raymond M, Rousset F (1995) GENEPOP (version 1.2): population genetics software for exact tests and ecumenicism. Journal of Heredity, 86: 248–249.

4. Wilson GA, Rannala B (2003) Bayesian inference of recent migration rates using multilocus genotypes. Genetics, 163: 1177–1191.

5. Aspi J, Roininen E, Kiiskilä J et al. (2009) Genetic structure of the northwestern Russian wolf populations and gene flow between Russia and Finland. Conservation Genetics, 10: 815–826.

6. Carmichael LE, Nagy JA, Larter NC, Strobeck C (2001) Prey specialization may influence patterns of gene flow in wolves of the Canadian Northwest. Molecular Ecology, 10: 2787–2798.

7. vonHoldt BM, Stahler DR, Smith DW et al. (2008) The genealogy and genetic viability of reintroduced Yellowstone grey wolves. Molecular Ecology, 17: 252-274.

8. vonHoldt BM, Stahler DR, Bangs EE et al. (2010) A novel assessment of population structure and gene flow in grey wolf populations of the Northern Rocky Mountains of the United States. Molecular Ecology, 19: 4412–4427.
